# Development of a multiplex droplet digital PCR assay for detection of enterovirus, parechovirus, herpes simplex virus 1 and 2 simultaneously for diagnosis of viral CNS infections

**DOI:** 10.1186/s12985-022-01798-y

**Published:** 2022-04-20

**Authors:** Xunhua Zhu, Pengcheng Liu, Lijuan Lu, Huaqing Zhong, Menghua Xu, Ran Jia, Liyun Su, Lingfeng Cao, Yameng Sun, Meijun Guo, Jianyue Sun, Jin Xu

**Affiliations:** 1 Department of Clinical Laboratory, Children’s Hospital of Fudan University, National Children’s Medical Center, Shanghai, China; 2 Shanghai Bio-Chain Biological Technology Co., Ltd, Shanghai, China; 3 Department of Pediatrics, Putuo Hospital, Shanghai University of Traditional Chinese Medicine, Shanghai, China

**Keywords:** Enterovirus, Parechovirus, Herpes simplex virus, Multiplex ddPCR, Viral CNS infections, qPCR, Quantification, Detection

## Abstract

**Background:**

Enterovirus (EV), parechovirus (HPeV), herpes simplex virus 1 and 2 (HSV1/2) are common viruses leading to viral central nervous system (CNS) infections which are increasingly predominant but exhibit deficiency in definite pathogen diagnosis with gold-standard quantitative PCR method. Previous studies have shown that droplet digital PCR (ddPCR) has great potential in pathogen detection and quantification, especially in low concentration samples.

**Methods:**

Targeting four common viruses of EV, HPeV, HSV1, and HSV2 in cerebrospinal fluid (CSF), we developed a multiplex ddPCR assay using probe ratio-based multiplexing strategy, analyzed the performance, and evaluated it in 97 CSF samples collected from patients with suspected viral CNS infections on a two-channel ddPCR detection system.

**Results:**

The four viruses were clearly distinguished by their corresponding fluorescence amplitude. The limits of detection for EV, HPeV, HSV1, and HSV2 were 5, 10, 5, and 10 copies per reaction, respectively. The dynamic range was at least four orders of magnitude spanning from 2000 to 2 copies per reaction. The results of 97 tested clinical CSF specimens were identical to those deduced from qPCR/qRT-PCR assays using commercial kits.

**Conclusion:**

The multiplex ddPCR assay was demonstrated to be an accurate and robust method which could detect EV, HPeV, HSV1, and HSV2 simultaneously. It provides a useful tool for clinical diagnosis and disease monitoring of viral CNS infections.

**Supplementary Information:**

The online version contains supplementary material available at 10.1186/s12985-022-01798-y.

## Background

Viral infections of the central nervous system (CNS) are potentially life-threatening and important causes of morbidity and mortality which take two primary forms: meningitis and encephalitis [1, 2]. Viral meningitis has symptoms similar to bacterial meningitis like fever, headache, vomiting, and signs of meningeal irritation [3]. Since the bacterial conjugate vaccine has greatly reduced the incidence of meningitis in children, the viral etiology has become increasingly prominent [4]. Encephalitis is a syndrome characterized by high fever, headache, vomiting, coma, convulsion, and mostly caused by neurotropic viruses [5].

The etiology of these viral CNS diseases is complicated. Enterovirus (EV) is the most common pathogen of viral meningitis in Western and South Asian countries. Herpesviruses, including herpes simplex virus 1 and 2 (HSV1 and HSV2) are also responsible for viral meningitis and encephalitis worldwide [6, 7]. In Australia, parechovirus makes almost equal contributions to CNS infection compared with EV [8]. The CNS of children is susceptible to EV and HPeV, and HSV encephalitis is common in both children and the elderly [9–11]. As these infections may lead to serious sequelae, prompt recognition and treatment are crucial for patients [6].

Quantitative real-time PCR (qPCR) is most commonly used in cerebrospinal fluid (CSF) detection. However, with relation to low viral-load body fluid such as serum and CSF, its accuracy, sensitivity, and repeatability are still not satisfying [12]. In addition, qPCR is susceptible to inhibitors and relies on standard calibrators which lead to variable qualitative and quantitative results among different laboratories. These limitations may compromise the timely diagnosis, early treatment, and antiviral therapy assessment in some viral infection cases [13]. And still, there is a large number of probable viral CNS infections not confirmed indeed due to the relatively limited diagnostic tools [2].

Digital PCR, a third-generation PCR technology, involves separately amplifying one or several nucleic acid molecules in small partitions to quantitate the initial targets [14, 15]. Droplet digital PCR (ddPCR) divides samples using water-in-oil droplets, then each droplet undergoes a PCR simultaneously, the number of positive and negative droplets is counted after the amplification and the copy number of targets is calculated based on Poisson statistics. Thus, the ddPCR approach has an absolute quantification capacity without using standard materials [16].

With the advantage of absolute quantitation without a standard curve and the potential of more accurate and sensitive end-point measurements, ddPCR has been applied to pathogens detection of infectious diseases like human immunodeficiency virus (HIV) [17–19], hepatitis B virus (HBV) [20], hepatitis C virus (HCV) [21] and viruses in aqueous humor [13].

Similar to qPCR, ddPCR can develop multiplex assays as well to reduce cost, labor, time, sample consumption, and accumulated pipetting inaccuracy [22]. The term “higher-order multiplexing” is used to describe the unique ability of dPCR to measure more targets precisely in one single reaction which overcomes the restriction of fluorescent detection channels [23, 24].

In clinical virology application, this capability has been described for typing of seasonal influenza virus and detecting the severe acute respiratory syndrome coronavirus 2 (SARS-CoV-2) with more than two targets by probe ratio- and amplitude-based multiplexing [22, 25]. As far as we are concerned, the multiplex detection of viruses in CSF using ddPCR has not been previously reported. In this study, we aimed to develop a quadruplex ddPCR assay using the probe ratio-based multiplexing strategy to detect and quantify EV, HPeV, HSV1, and HSV2 simultaneously in CSF and to diagnose the viral CNS infections.

## Methods

### Primers and probes

Primers and probes targeting HSV1, HSV2, and HPeV were reported previously [26, 27]. Primer–probe set targeting highly conserved 5’ untranslated region (5’UTR) of EV was designed from an alignment of GenBank sequences using Primer Premier 5.0 (Primer Biosoft International, USA). An in-silico analysis using the tool of Primer-BLAST in NCBI was performed. All primers and probes are highly specific to the different types of virus sequences in the Nucleotide database of NCBI and synthesized by Sangon, China. EV probe was labeled with a fluorophore of FAM. HSV1 was labeled with VIC. HPeV and HSV2 were labeled with FAM and VIC. The sequences of the primers and probes and their optimized concentrations were given in Table 1.

**Table 1:** Sequences of primers and probes

Primer/probe	Sequence (5′–3′)	Concentration, nmol/L		Refs.
		Simplex ddPCR	Quadruplex ddPCR	
EV-for^a^	GGTGTGAAGAGCCTATTGAGCT	400	400	In house
EV-rev^b^	GAAACACGGACACCCAAAGTAGT	400	400	
EV-probe	FAM-TCCTCCGGCCCCTGAATGCGGCTAATCC-BHQ1	200	200	
HSV2-for	CGCTCTCGTAAATGCTTCCCT	400	400	[23]
HSV2-rev	TCTACCCACAACAGACCCACG	400	400	
HSV2-probe	FAM-CGCGGAGACATTCGAGTACCAGATCG-BHQ1	–	100	
	VIC-CGCGGAGACATTCGAGTACCAGATCG-BHQ1	200	100	
HPeV-for	GTGCCTCTGGGGCCAAAAG	400	400	[24]
HPeV-rev	TCAGATCCATAGTGTCGCTTGTTAC	400	400	
HPeV-probe	FAM-ACGGGTACCTTCTGGGCATCCTTCG-BHQ1	200	50	
	VIC-ACGGGTACCTTCTGGGCATCCTTCG-BHQ1	–	150	
HSV1-for	CGGCCGTGTGACACTATCG	400	400	[23]
HSV1-rev	CTCGTAAAATGGCCCCTCC	400	400	
HSV1-probe	VIC-CCATACCGACCACACCGACGAAC-BHQ1	200	400	

^a^Forward primer

^b^Reverse primer

### Test materials, nucleic acid extraction, and reverse transcription

EV strain and positive clinical specimens of HSV1/2 and HPeV confirmed by sanger sequencing were used for assay development and performance evaluation. The viral RNA and DNA were simultaneously extracted and purified with a kit on an automated extraction machine both from Tianlong, China. Reverse transcription was performed then on T100 thermal cycler (Bio-Rad, USA) with a mixture of 8 μl of EV or HPeV nucleic acid, 4 μl of 5 × PrimeScritBuffer, 2 μl of Random 6mers, 1 μl of PrimeScript RT Enzyme Mix I, and 5 μl of nuclease-free water (TaKaRa, Japan). The reaction was carried out at 37 °C for 30 min and 85 °C for 5 s.

### Simplex and quadruplex ddPCR

The QX200 Droplet Digital PCR System (Bio-Rad, USA) was used for the assay development of simplex and quadruplex ddPCR. For simplex ddPCR, the reaction mixture consisted of 10 µL 2 × ddPCR Supermix for probes (No dUTP), a pair of specific primer–probe set including 1 µL primers and 1 µL probes at a final concentration of 400 and 200 nM, respectively, 2 µL cDNA/DNA, and 6 µL nuclease-free water to 20 µL.

As the QX200 ddPCR System has only two detection channels, two targets at most can be quantified within a single reaction in general settings. In this study, we adopted the strategy of probe ratio-based multiplexing to develop a quadruplex ddPCR assay. Based on the simplex ddPCR, 4 sets of primers and probes were added in one single reaction to develop the quadruplex ddPCR. For EV, 200 nmol FAM-labeled probe was added; for HSV1, 400 nmol VIC-labeled probe was added; for HSV2, 100 nmol FAM-labeled and 100 nmol VIC-labeled probe were added; for HPeV, 50 nmol FAM-labeled and 150 nmol VIC-labeled probe were added. All the primers were added at a final concentration of 400 nM. A reaction mixture of 20μL was composed of 10μL 2 × ddPCR Supermix for probes (No dUTP, Bio-Rad), 1 μL primer mix, 1 μL probe mix, 2 μL cDNA/DNA, and 6μL nuclease-free water. The 20 μL reaction mix and 70 μL droplet generation oil were added to a droplet generation cartridge (DG8, Bio-Rad) to generate approximately 20,000 nanoliter-sized droplets in a droplet generator (QX200 system, Bio-Rad). Then 40 μL emulsion made of water-in-oil droplets was transferred to a 96-well plate and sealed with a pierceable aluminum foil using a PX1 PCR plate sealer (Bio-Rad) at 180 °C for 5 s. Samples were amplified in the T100 Thermal Cycler (Bio-Rad) according to the following steps: 96 °C for 10 min (enzyme activation), 40 cycles of 98 °C for 30 s (denaturation) and 55 °C for 60 s (annealing), and 60 °C for 2 min. The amplified droplets were detected in a Droplet Reader (Bio-Rad).

### Performance evaluation of quadruplex ddPCR

The cDNA/DNA mixture of four targets from EV stock and positive clinical specimens confirmed by Sanger sequencing after reverse transcription was used to evaluate the performance of the Quadruplex ddPCR assay. The copy number of each target was determined by simplex ddPCR and then diluted to the corresponding concentrations we need.

The analytical sensitivity or limit of detection (LOD) was assessed using dilutions of the cDNA/DNA mixture in DNase- and RNase-free water with concentrations of 50, 10, 5, and 2 copies per reaction. Each concentration was detected in 20 replicates. The LOD for each target was defined as the lowest dilution where at least 19 replicates still produced a positive result [28]. Based on the LOD assessment, the limit of quantification (LOQ) was defined as the lowest concentration at which the CV (coefficient of variation) of all replicates was below 25% [28].

Another group of tenfold serial dilutions of nucleic acid mixture throughout 2000 to 2 copies per reaction was used to evaluate the dynamic range. Each concentration was run in 3 replicates.

For the limit of blank (LOB), 10 mNGS-negative (metagenomic next-generation sequencing) CSF samples and 4 no-template controls (NTC) were used. The number of positive droplets ≥ LOB was considered positive.

The analytical specificity of the assay was determined by performing cross-reactivity tests in other pathogens susceptible to CNS infections as well including varicella-zoster virus, Epstein-Barr virus, cytomegalovirus, human herpesvirus 6 (positive clinical specimens confirmed by mNGS), *Streptococcus aureus* (ATCC25923), *Pseudomonas aeruginosa* (ATCC27853), *Streptococcus pneumonia* (ATCC49619), *Escherichia coli* (ATCC25922), *Staphylococcus epidermidis*, *Staphylococcus hominis*, *group B streptococcus*, *Staphylococcus haemolyticus*, *Klebsiella pneumonia* and *Acinetobacter baumannii* (clinical isolation strains identified by mass spectrometry). After DNA extraction, the concentration of these strains was measured by NanoDrop2000 (Thermo Scientific, USA).

### Validation of quadruplex ddPCR using clinical specimens

A total of 97 CSF samples from patients suspected of viral CNS infections were collected for assay validation from May 2020 to December 2021 and stored at -70℃ before use. These samples along with 10 HPeV-positive stool samples and 2 HSV2-positive secretion samples were simultaneously detected by qPCR/RT-qPCR and ddPCR methods. When testing patient CSF samples, multiplex assays always included a positive control that was a nucleic acid mixture of four viruses to assure no abnormalities in cluster distribution thus no misidentification for each virus, especially for HSV2 and HPeV. The commercial qPCR/RT-qPCR kits (HSV1 from Bai Tai, HSV2 from Da-An, EV from Mo Le, and HPeV from Shuo Shi, China) were used to detect these clinical samples following the manufacturer's instructions on an Applied Biosystems 7500 system (Thermo Fisher, USA). Among the kits, EV, HSV1, and HSV2 are approved by the Chinese FDA. Since the HPeV is not commonly and routinely detected in clinical laboratories in China, the assay provided for China CDC for surveillance and research is used. The results of the two methods were compared. This study was approved by the ethical committee of the Children’s Hospital of Fudan University (2021276).

### Data analysis

The results and data of ddPCR were generated and analyzed by QuantaSoft software version 1.7.4.0917 or QuantaSoft™ Analysis Pro version 1.0.596 when templates were relatively high (Bio-Rad). Linear regressions were done using the GraphPad Prism7 software and CV for LOQ was calculated by Excel.

## Results

### Simplex ddPCR

**Fig. 1:**
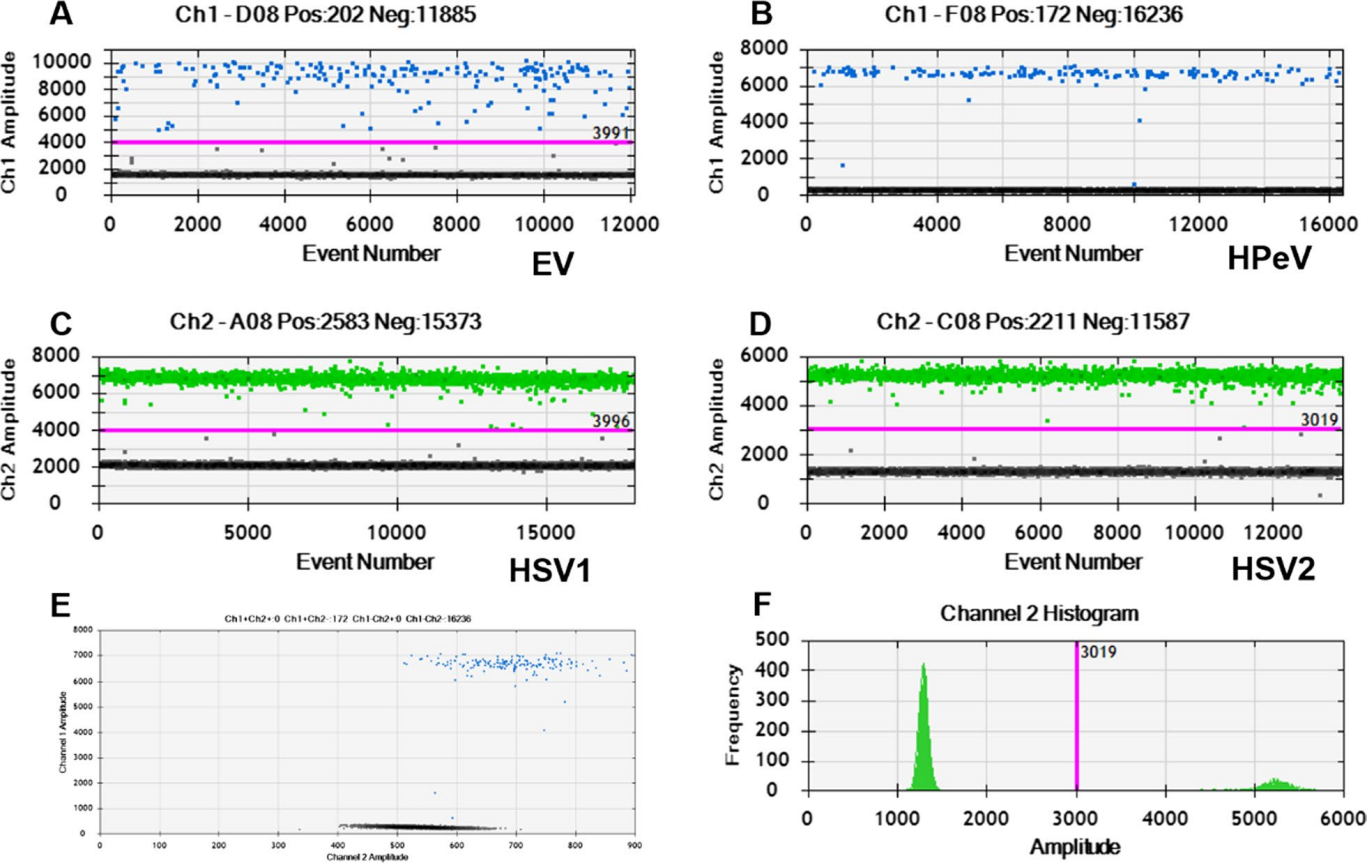
The results of simplex ddPCR assay. **A**, **B**, **C**, and **D** are one-dimensional (1D) plots of the EV, HPeV, HSV1, and HSV2 respectively. The x-axis is event number and the y-axis stands for fluorescent amplitude. **E** is a representative two-dimensional (2D) plot. F is a typical histogram of a simplex assay

Each simplex assay could produce a positive result with an obvious amplitude difference between positive and negative droplets as shown in Fig. 1. Both the EV and HPeV were FAM-labeled hence present in Channel 1 (Ch 1) with blue positive droplets above the threshold line (red line). Gray negative droplets with background amplitude were below the line. The HSV1 and HSV2 were labeled with VIC hence present in Channel 2 (Ch 2) with green positive droplets.

### Droplet separation of quadruplex ddPCR

**Fig. 2:**
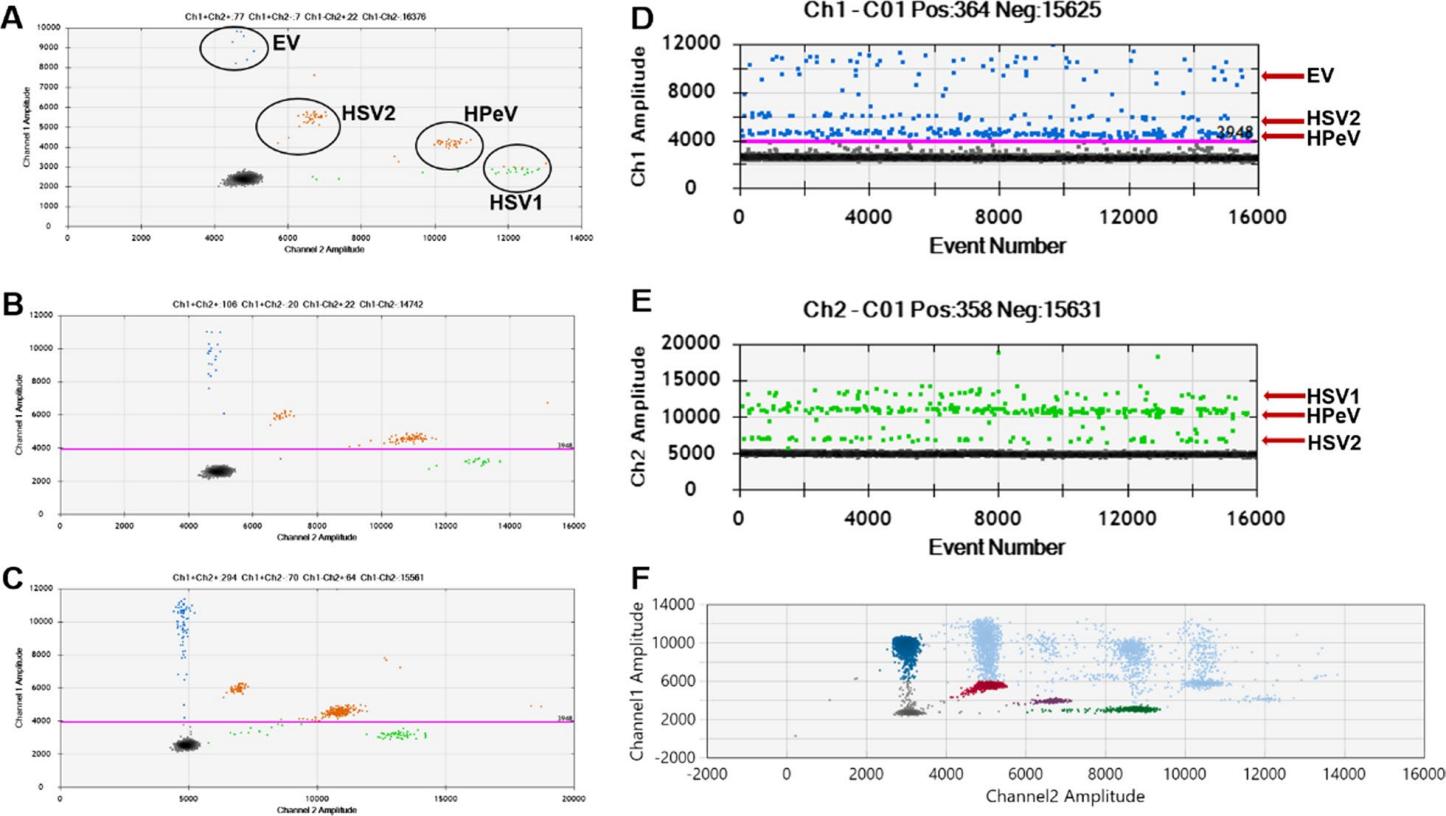
Quadruplex ddPCR assay droplet separation results. **A** is a two-dimensional (2D) amplitude result of quadruplex ddPCR assay. The x-axis is the fluorescence amplitude at Channel VIC (Channel 2, Ch2) and the y-axis stands for channel FAM (Channel 1, Ch1). **A**, **B**, and **C** are 2D plots using different concentrations of nucleic acid mixtures. **D** and **E** are two typical 1D plots of quadruplex ddPCR assay and generate from the same reaction of **C**. The positive droplets located at specific areas represent different positive targets as indicated by the red arrow. F is a representative result of quadruplex ddPCR assay performed on a 10^5^ dilution (high concentration) of the nucleic acid mixture. Blue droplets: positive for EV; red droplets: positive for HSV2; purple droplets: positive for HPeV; green droplets: positive for HSV1; grey droplets: negative; the others (light blue droplets) are positive for one FAM- and one VIC-labeled target at least

The test materials described above were used to develop the quadruplex ddPCR. A total of 4 primer–probe sets labeled with different fluorophores and in optimized concentrations were added to a single multiplex ddPCR reaction. By adding appropriate template concentrations (not very high), 5 droplet clusters could be observed as shown in Fig. 2A. A quadruple negative cluster was located at the left bottom; the EV-positive cluster added with 200 nM FAM only was found in channel 1 directly above the quadruple negative; the HSV1-positive cluster added with 400 nM VIC only was observed in channel 2 right next to the quadruple negative; the HSV2-positive both added with 100 nM FAM and 100 nM VIC was hence read between the two channels at an about > 45° angle from the quadruple negative; added with 50 nM FAM and 150 nM VIC, the HPeV-positive was presented at a < 30° angle from the quadruple negative. The fluorescence amplitude of HSV2 in the x-axis is about 7000 and the y-axis is about 6000. The fluorescence amplitude of HPeV in the x-axis is about 11,000 and the y-axis is about 5000. All the clusters were separated distinctly and maintained at the same position even changing the concentration of templates (Fig. 2A–C). When more concentrated templates were used, 16 (2^4^) clusters were produced at most in the 2D amplitude. The reason is that there were two or more targets present in one droplet at the same time, so the fluorescence increased accordingly (Fig. 2F).

### Performance of the quadruplex ddPCR assay

**Fig. 3:**
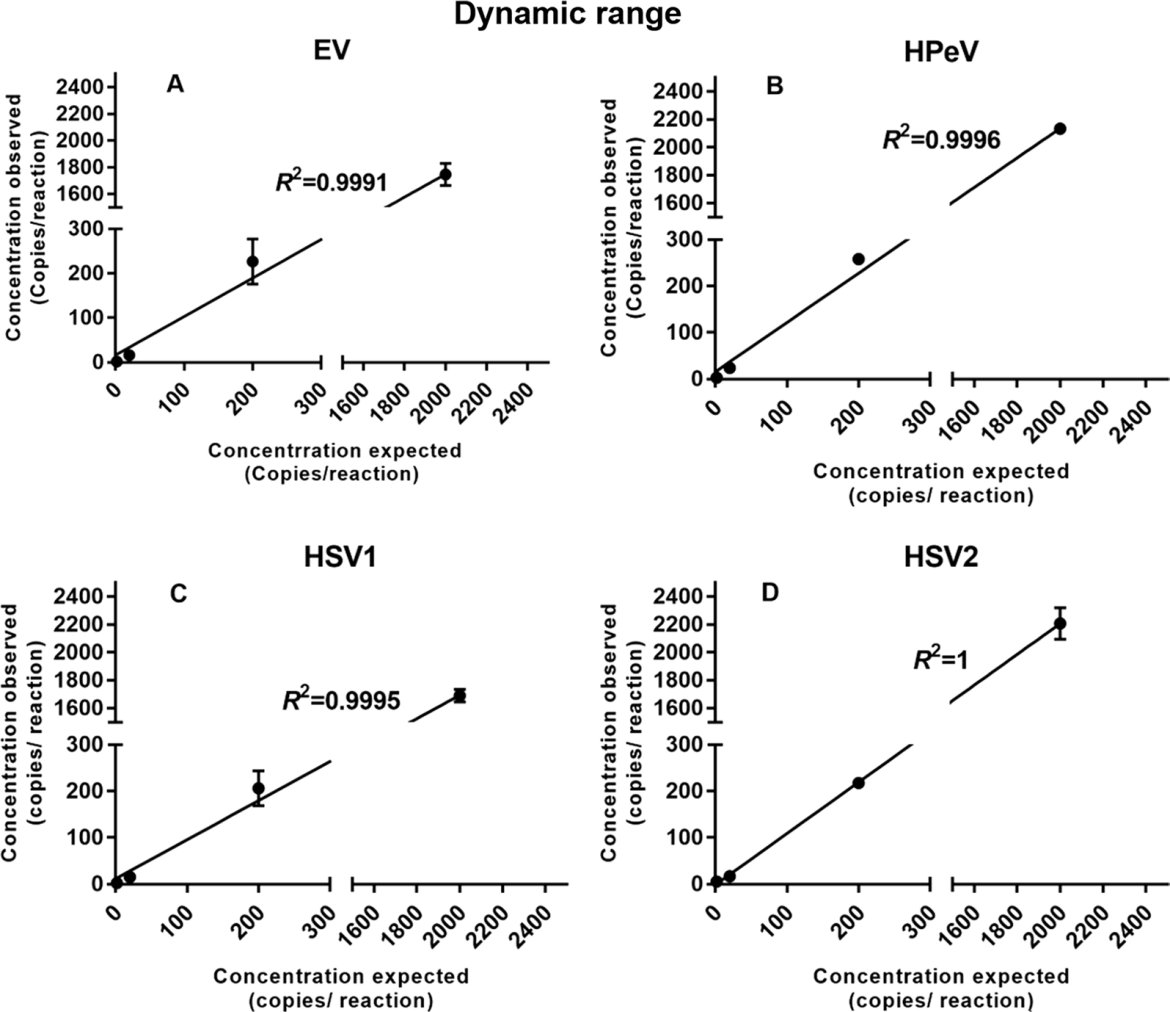
Dynamic range for the four viruses. Three replicates were performed for each dilution ranging from 2000 to 2 copies per reaction

The LODs of the quadruplex ddPCR for EV, HPeV, HSV1, and HSV2 were 5, 10, 5, and 10 copies per reaction, respectively. The LOQ values of our four targets defined as the lowest concentration that can be quantified with a CV ≤ 25% were 10, 10, 50, and 10 copies per reaction, respectively (Table 2). All the LOB was determined to be 1 positive droplet (Additional file 1: Fig. S1). No cross-reactions were observed with other common pathogens not included in our panel which is also related to CNS infections described above, indicating that the quadruplex ddPCR assay had a great analytical specificity for the detection of our targeting viruses (Additional file 1: Fig. S2). The dynamic range was turned out to be at least four orders of magnitude spanning from 2000 to 2 copies per reaction. The linear regression of each virus between expected and observed concentrations were made and the *R*^2^ was as follows: EV: 0.9991; HPeV: 0.9996; HSV1: 0.9995; HSV2: 1, which showed a good linear relationship and hence a great capacity of quantification in this range of dilutions at least (Fig. 3).

**Table 2:** Limit of detection and quantification

	Virus			
	EV	HPeV	HSV1	HSV2
Mean^a^	13.43 (10^b^)	13.25 (10)	37.80 (50)	12.68 (10)
SD	2.70	3.23	7.76	3.07
CV%	20.14	24.38	20.52	24.17
LOQ^c^	10	10	50	10
LOD^d^	5	10	5	10

SD, standard deviation; CV, coefficient of variation

^a^Average of twenty replicates, in copies per reaction

^b^Expected concentration determined by simplex ddPCR

^c^Limit of quantification

^d^Limit of detection

### Clinical specimen detection for validation

Of the 119 clinical specimens tested, the 10 HPeV-, 10 HSV1- and 2 HSV2-positive samples confirmed by qPCR/RT-qPCR previously produced positive results by quadruplex ddPCR too. The other 97 CSF specimens were detected by qPCR/RT-qPCR and quadruplex ddPCR simultaneously. The results of the 97 samples were fully consistent using the two assays. EV was found in most cases (15/97, 15.46%). HPeV and HSV1 were found in 3 (3.09%) and 2 (2.06%) cases, respectively. No HSV2 was found (Table 3).

**Table 3:** Diagnostic performance of quadruplex ddPCR and qPCR/RT-qPCR in detecting clinical CSF samples

Virus	No. of specimens by quadruplex ddPCR Versus qPCR/RT-qPCR		Detection rate (%)
	+/+^a^	−/−^b^	
EV	15	82	15.46
HPeV	3	94	3.09
HSV1	2	95	2.06
HSV2	0	97	0

^a^Positive in two assays

^b^Negative in two assays

All but 2 children with positive CSF were born at term. 55% of patients were < 28 days. The median length of hospitalization was 11 days ranging from 1 to 43 days. 13 patients were diagnosed with meningitis, 6 with encephalitis, and 1 with meningoencephalitis (Additional file 1: Table S1).

## Discussion

Viral CNS infection, which is characterized by fever, headache, seizures, vomiting, and altered mental status caused by various viruses is being increasingly serious and prominent since the popularity of vaccines and the increase of immunodeficient patients [3–5]. EV, HPeV, HSV1, and HSV2 have become the main causes around the world [6, 8]. However, due to the characteristic of low viral loads in CSF, the detection rate of pathogens is underestimated and the definite pathogen diagnoses are insufficient [2]. Moreover, it’s reported that the viral loads can assess disease severity and predict clinical outcomes [9]. Thus, a highly sensitive approach with strong quantitative ability is needed. Compared to qPCR, digital PCR is more accurate in low nucleic acid samples and resistant to PCR inhibitors [16]. Compared with the simplex assay, multiplex ddPCR can save cost, labor time, and sample given to the few CSF volumes in children, especially in neonates.

In this study, we have developed and evaluated a quadruplex ddPCR. There are two main strategies when related to multiplex ddPCR assay development with more than two targets: probe ratio-based multiplexing with three targets and amplitude-based multiplexing with four or more targets [25]. In our study, we innovatively adopted the multiplex probe ratio strategy with four different targets. In this way, there are two distinct single-positive droplet clusters (HSV2-, HPeV-positive, Fig. 2) between the X and Y axes. The stable separate clusters in the 2D plot (Fig. 2) proved that we have developed a quadruplex ddPCR distinguishing four targets successfully with a broad range of linearity and good sensitivity (Fig. 3).

LOB is an important parameter for a digital PCR assay which defines the qualitative results of negative or positive. The determination of LOB depends on the accumulating results by testing a large number of negative samples. Different assays have different LOB values due to the difference in templates, probe concentration, and reaction conditions. Researchers are supposed to determine the LOB themselves. In our study, the LOB of each virus was one positive droplet. This means the false-positive droplets are few in our method.

In our study, the results of qPCR and ddPCR are completely consistent, which means our quadruplex ddPCR assay has great sensitivity comparable to commercial qPCR/qRT-PCR kits but more detectable targets and powerful quantitative quality, especially in low concentration samples. EV was the main cause of viral CNS infection (15/97, 15.46%), identical to other studies in China [29, 30]. HPeV was found in 3 cases. Notably, it’s reported that HPeV especially the HPeV3 associated with severe cases is as common as EV in CNS infection enjoying similar symptoms [31]. So, the detection and identification of these two viruses are very important and urgent. However, few laboratories include HPeV in routine testing items in China. Our multiplex ddPCR assay is a useful tool for confirmatory and differential diagnosis of these two viruses. HSV1 was found in 2 cases and encephalitis caused by HSV1 is generally severe [32].

There were several limitations in our study. First, HSV2 was not found due to the limited CSF sample numbers. So, we have tested 2 HSV2-positive secretion samples to alternately evaluate the clinical diagnosis capacity of HSV2. Second, because of the limited detection channels, internal control was not added to the multiplex assay. Third, we determined the LOB using 10 mNGS-negative CSF samples because the CSF of healthy population couldn't be obtained.

In the future, more CSF samples should be detected to further improve the method and apply it to clinical use. New panels focusing on other pathogens in CSF or bloodstream infections which is also featured by low concentration of pathogens could be developed using the multiplex ddPCR approach. In addition, we will continue to focus on increasing the number of detection targets and then incorporate more pathogens and the internal control into our assay, optimizing analytical performance, and further improving the sensitivity and accuracy of the multiplex ddPCR.

## Conclusion

In conclusion, we have developed a sensitive and robust quadruplex ddPCR assay which could detect EV, HPeV, HSV1, and HSV2 simultaneously. It provides a useful tool for clinical diagnosis and disease monitoring of viral CNS infections.

## Supplementary Information

**Additional file 1.** **Figure S1:** The result of limit of blank (LOB) from 10 mNGS-negative CSF samples and 4 no-template controls. **Figure S2:** The result of analytical specificity from 14 pathogens and positive control. **Table S1:** Demographic and clinical characteristics of patients with positive specimens.

## Data Availability

Original data and materials are available from the corresponding author.

## Abbreviations

CNS: Central nervous system
ddPCR: Droplet digital PCR
EV: Enterovirus
HSV1: Herpes simplex virus 1
HSV2: Herpes simplex virus 2
HPeV: Parechovirus
CSF: Cerebrospinal fluid
qPCR: Quantitative PCR
RT-qPCR: Reverse transcription quantitative PCR
5’UTR: 5’ Untranslated region
LOD: Limit of detection
LOQ: Limit of quantification
CV: Coefficient of variation
SD: Standard deviation
