# Supplementary material for: Development of a multiplex droplet digital PCR assay for detection of enterovirus, parechovirus, herpes simplex virus 1 and 2 simultaneously for diagnosis of viral CNS infections
Source: Virol J. 2022 Apr 20;19:70. doi: 10.1186/s12985-022-01798-y (PMC9020426; doi:10.1186/s12985-022-01798-y)
Supplement: Supplementary file 1 — Additional file 1. Figure S1: The result of limit of blank (LOB) from 10 mNGS-negative CSF samples and 4 no-template controls. Figure S2: The result of analytical specificity from 14 pathogens and positive control. Table S1: Demographic and clinical characteristics of patients with positive specimens. [file 12985_2022_1798_MOESM1_ESM.docx]

**Figures**


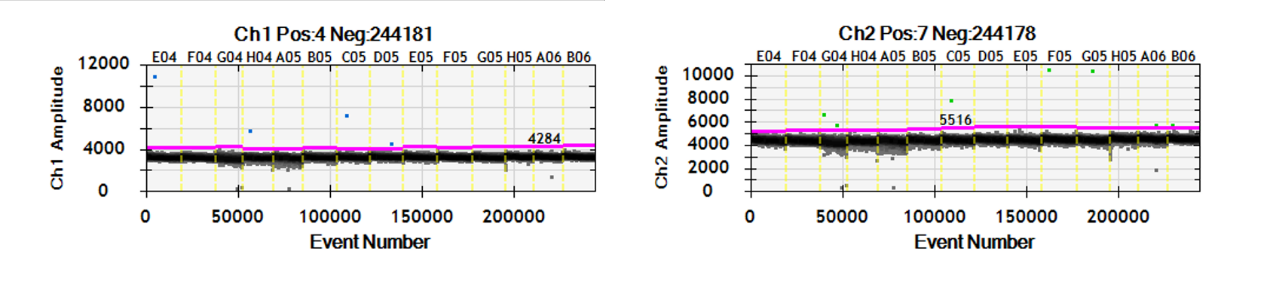
**Fig. S1** The result of limit of blank (LOB) from 10 NGS-negative CSF samples and 4 no-template controls.


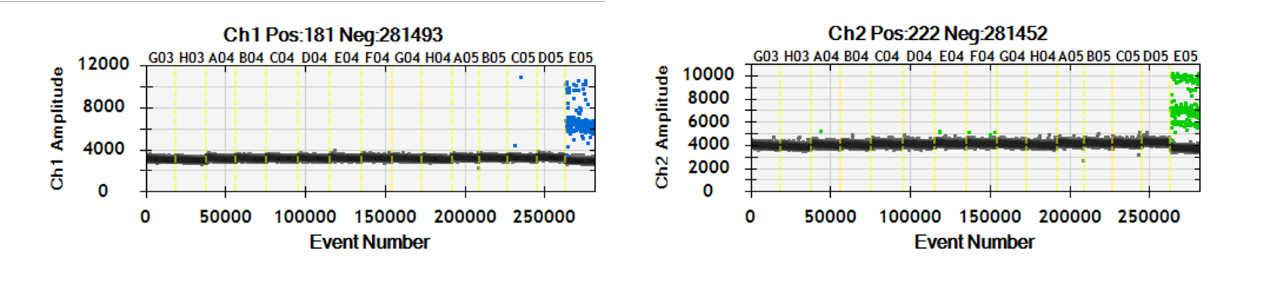


**Fig. S2** The result of analytical specificity from 14 pathogens and positive control (E05).

**Table**

**Table S1.** Demographic and clinical characteristics of patients with positive specimens

| Characteristics | N (%) |
| --- | --- |
| Male sex | 10 (50) |
| Median of age (range) | 26 days (4 days to 13 years) |
| <28 days | 11 (55) |
| 28 days-1 year | 3 (15) |
| 1-6 years | 3 (15) |
| 6-13 years  Median length of hospitalization (range) | 3 (15)  11 days (1 day to 43 days) |
| Type of infection |  |
| Meningitis | 13 (65) |
| Encephalitis | 6 (30) |
| Meningoencephalitis | 1 (5) |
| Symptoms and signs |  |
| Fever | 18 (90) |
| Headache | 5 (25) |
| Vomiting | 9 (45) |
| Seizures | 4 (20) |
| Diarrhea | 4 (20) |
| Neck rigidity | 3 (15) |
| Brudzinski’s sign | 2 (10) |
| Skin rash | 6 (30) |
| Preterm birth | 2 (10) |
| Use of antibiotics | 18 (90) |
| Use of acyclovir | 8 (40) |
